# A Novel Topology Link-Controlling Approach for Active Defense of Nodes in Networks

**DOI:** 10.3390/s17030553

**Published:** 2017-03-09

**Authors:** Jun Li, HanPing Hu, Qiao Ke, Naixue Xiong

**Affiliations:** 1 School of Automation, Huazhong University of Science and Technology, Wuhan 430070, China; hphu@mail.hust.edu.cn; 2 Department of Computer Science, Hubei University of Technology, Wuhan 430070, China; 3 Department of Electrical Engineering, University of North Texas, Denton, TX 76203, USA; Qiao.Ke@unt.edu; 4 Department of Mathematics and Computer Science, Northeastern State University, Tahlequah, OK 74464, USA; nxiong@coloradotech.edu

**Keywords:** link-controlling, copula, resource protection, minority game

## Abstract

With the rapid development of virtual machine technology and cloud computing, distributed denial of service (DDoS) attacks, or some peak traffic, poses a great threat to the security of the network. In this paper, a novel topology link control technique and mitigation attacks in real-time environments is proposed. Firstly, a non-invasive method of deploying virtual sensors in the nodes is built, which uses the resource manager of each monitored node as a sensor. Secondly, a general topology-controlling approach of resisting the tolerant invasion is proposed. In the proposed approach, a prediction model is constructed by using copula functions for predicting the peak of a resource through another resource. The result of prediction determines whether or not to initiate the active defense. Finally, a minority game with incomplete strategy is employed to suppress attack flows and improve the permeability of the normal flows. The simulation results show that the proposed approach is very effective in protecting nodes.

## 1. Introduction

The rapid development of virtual machine and cloud computing technologies has led to a serious situation in network security. One of the major problems is that network bandwidth has been raised from gigabit to ten-gigabit. However, the existing network devices lack adequate performance and scalability to deal with these changes. Another problem being faced in technology development is that network defense systems are unable to protect network resources during attacks. According to the latest report provided by the global content delivery network (CDN) service provider, the number of distributed denial of service (DDoS) attacks has increased by 118% from the previous year, and will continue to increase in 2016 [1]. It happened the first time when the proportion of 10 gigabits per second (Gbps) traffic in DDoS attacks was revealed to be more than 20%, which indicates the expanding trend of DDoS attacks. Moreover, the strength of these attacks can be multiplied through several freely-available attack tools on the Internet and virtual machine technology.

The main objective of network security is to maintain the availability of network resources during a variety of network behaviors—especially attack behaviors. Major network resources include bandwidth, memory, delay, throughput, CPU, and disk resources, etc. A DDoS attack can make the victim’s resources unusable for some time or even cause a permanent cessation by sending a substantial amount of packets [2]. On the other hand, a high volume of traffic is also generated in the form of legitimate requests during flash events (FE), which need to be serviced by provisioning extra resources. DDoS attack traffic and FE traffic share a number of similar characteristics [3]. Thus, detecting known and unknown DDoS attacks and FE traffic is becoming one of the most difficult problems. Existing intrusion detection systems, firewalls, and other defense techniques are unable to address the network security issues—especially the DDoS attacks. DDoS attacks generate a scarcity of network resources. Thus, in an unsecure network environment, resources must be protected under tolerance invasion.

The main goal of our research is the active protection of resources in an intrusion tolerance network. It is well known that different networks have different requirements. However, when a network challenge arises, it makes more sense for networks to quickly reduce threats than to precisely filter malicious packets. It is better to detect the threat of various attacks, including FE, as soon as possible. Early detection of an attack ensures that defense devices have enough time to deal with threats. Early detection can become not only a method of discriminating the attack, but also an important standard of defensive measures from the start. Therefore, building an appropriate resource prediction model has important theoretical value and practical significance.

On the other hand, one of the major features of DDoS attacks is the attack scale. According to the statistics of the flow behavior, a large amount of attack traffic occupies most of the whole traffic during the invasion period, and normal behavior traffic surely becomes the minority. That means some flows—Such as normal flows, which account for a small proportion in the total flows—Are harmless. Many flow behaviors compete for limited network resources, and make decisions by their respective strategy. This generates a scenario that is similar to the management of financial markets or network congestion. To solve such problems, new methods—Especially minority game models—Have been applied in the research field. Here, a new approach involves the improvement of the minority behaviors for their chances of survival and the depression of the majority behaviors to protect the network.

**Figure 1:**
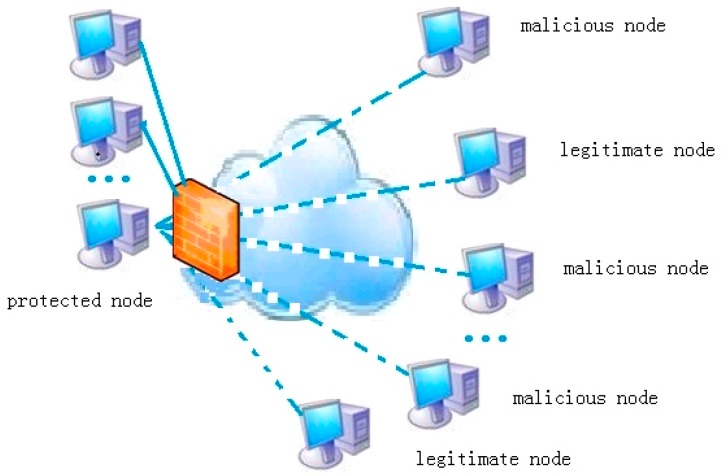
Topology control in emerging sensor networks.

Recent studies [4,5,6,7,8,9,10,11,12,13] proposed some methods to filter malicious packets and verify intruder attempts by monitoring the impact of user behavior on the service level agreement, the network attacks are more towards a specific computer than the entire network. Therefore, we need to monitor some computers whose conservation is given priority status. It is assumed that one server is a protected node, all of the links between the node and all external ones form a generalized star network according to different IPs, and a large number of normal network behaviors and malicious behaviors are hidden in these links. With the change of the traffic in the links, the resources of the server are affected. As shown in Figure 1, numerous links can be divided according to IP address, port, and protocol number, and each packet is transmitted through a link to the protected nodes. The generalized logical links are combined with a larger network. Virtual links contain many malicious and legitimate external nodes, and link-controlling would provide a new idea to protect the node. When the resources of the key node are short, we can interrupt the flow of certain links through the firewall or switch; that is, the link control can achieve the purpose of protecting the arbitrary network node.

In this paper, we attempt to analyze the relationship between the traffic on the firewall and the server’s resources, and build a model for the prediction of peak resources. Then, the result of prediction determines the decision to start the active defense. Finally, a minority game approach is proposed to mitigate attacks in real-time environments and prevent DDoS attack (forged) packets from reaching the target, while allowing genuine packets.

Our primary aim is to construct a defense mechanism to ensure the survival of the network after the occurrence of threats. The specific contributions of this paper include:

- A non-invasive method of deploying virtual sensors is proposed, which use the resource manager of each monitored node as a sensor;
- An early prediction model of node resource risk on the gateway is proposed. This model predicted the level of the resources of a protected node from the traffic on the firewall so as to provide the basis for the defense measures to start; and
- The proposed method of mitigating attacks in real-time environments to prevent malicious packets reaching the target, while allowing legal packets.

The rest of this paper is organized as follows: A brief overview of the related works is presented in Section 2. Section 3 discusses the theoretical framework and architectural design of the proposed approach. The simulation results and performance analysis are presented in Section 4. Finally, Section 5 concludes the paper and discusses some future research directions.

## 2. Related Work

The work in this area can be classified into three categories that are based upon prediction (detection), active defense, and the minority game.

The detection category of research uses anomaly-based detection methods to detect DDoS attacks. Some researchers focused on exploiting entropy-based aspects to detect DDoS attacks and neutralize the effect of an attack [14]. Xiao et al. presented an effective detection approach based on K-nearest neighbors traffic classification with correlation analysis to detect DDoS attacks [15]. Alenezi derived an optimal marking scheme called uniform probabilistic packet marking (UPPM) to identify the attackers so that their attack traffic can be blocked at source [16]. Saied et al. selected an artificial neural network (ANN) algorithm to detect DDoS attacks based on specific characteristic features (patterns) that separate DDoS attack traffic from genuine traffic [17]. However, these methods need a lot of training samples for learning and could not respond well to novel attacks. Here, traffic is the main object of their studies, and they consider the relation between the flows and the network. However, distinguishing legitimate packets of normal traffic and peaks of abnormal traffic sent by compromised hosts to their victims is a challenging task. 

The second category of research in the field uses active defense to neutralize attack effects. Currently, many researchers have proposed numerous DDoS defense methods. Beitollahi et al. proposed a cooperative defense mechanism between a victim’s server and two types of upstream routers to audit and control connections [18,19]. Confidence-Based Filtering (CBF) is another packet technique that calculates the score of every packet according to packet flow statistics of extracting attribute pairs [20]. Vissers introduced a model of extracting connection features on the application layer to filter flooding attacks [21]. Spyridopoulos et al. modeled a DDoS attack as a one-shot, non-cooperative, zero-sum game in terms of the cost to perform an attack, the number of attacking nodes, malicious traffic probability distributions, and their parameters [22,23,24]. Bedi et al. presented a deterministic fair sharing (DFS) model which uses the concept of weighted fair share (WFS) that allows it to dynamically self-adjust the router buffer usage based on the current level of congestion, while aiding in identifying malicious flows [25]. Adamsky presented an experimental analysis of bandwidth attacks against different choking algorithms in the Bit-Torrent seed states, and the author reported the damage caused by the proposed attack in two different environments. He proposed a novel choking algorithm which is immune against bandwidth attacks and a countermeasure against the revealed attack. These works were focused on identifying malicious flows from connections, but neglected how many normal flows can pass, and did not mention whether the result of the action processing can ensure the network gets into the security state.

The third category of related work is based on a minority game (MG) model. The MG model has recently gained significant attention in many application areas. Chen built an evolutionary mixed-game model to predict the Shanghai Index by adding the abilities of strategy evolution to agents [26]. Chau constructed a minority-game-like economic model which proved that the level of cooperation in the networked minority game differs remarkably from the original minority game and prediction of the crowd–anticrowd theory [27]. Zu proposed a congestion control model based on a minority game with local information [28]. The model adjusts the transmission rate based on the game prediction result, improves the links utilization, and rapidly reduces the congestion. The proposed method is based on the Transmission Control Protocol (TCP) sliding window protocol, which makes it difficult to modify the protocol and apply the model. The application of this method to the router hardware provides a useful reference for us.

Despite the many solutions, the problem is hardly tackled. Most of the solutions lack the capability to cope with unknown attacks, whereas some solutions have high computational complexity. However, active defense-based solutions are general and quick methods to mitigate threats which require a deep analysis of the protocol and packet information. These methods are significantly affected by the learning knowledge base.

## 3. The Proposed Model

**Figure 2:**
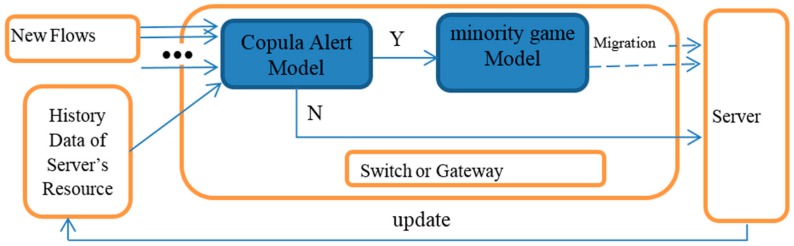
Schematic representation of the proposed model.

In this section, we present the description of the proposed model. As shown in Figure 2, the new approach comprises two main models. A copula alert model uses a copula function for predicting the peak of a resource. If predictions do not reach low thresholds, then net flows can be transferred, directed through typed gateways. The alert system continues updating the new observed server’s resource data consistently. The other model is a minority game model of mitigating the risks. If an alert is issued, then this model will block the flows according to the game results at the gateway. The collaborative working principle of two modules is schematically represented in Figure 2.

### 3.1. Early Alert Model 

#### 3.1.1. Single Resource Early Warning Model

Many methods are available to only predict variations of risks against resources, such as the moving average method and the neural network-based prediction method. These methods have their pros and cons. More importantly, these prediction modules are usually implemented at the protected nodes. As a result, little time is left to make a response when the risk is predicted at the node. However, in our proposed method, the prediction module is installed in the gateway before the flow entering the node, and the resources at the node are predicted based on the flow of the gateway. The retransmission time is set up according to the retransmission timing rule for each type of packet in the network protocols (e.g., RFC6298 in [29]). For example, retransmission time of less than 200 ms is allowed in TCP. We can collect packets of nodes at the gateway during this time period for prediction and filtering.

Certainly, there is some intrinsic correlation between network resources. However, in previous studies, prediction of a variable often used a single historical time series, and ignored the correlation of multiple variables. However, in some cases (such as hard real-time capture), the peak of a variable can be predicted through another variable, which offers a new method for data prediction.

CPU and memory are important resources in the network. In general, a flow behavior cannot be executed without these resources, and network behavior is usually hidden in the traffic. Therefore, we selected the flow, CPU, and memory resources as the main research objects, and made predictions through their relationships.

Research has shown that there is a kind of complex correlation among network resources. In some sense, the flow behavior’s cause and effect changes the resources, but building a joint distribution function composed of multiple random variables of network resources is a complex problem. The ordinary linear correlation coefficient cannot reflect the characteristics of the correlation between the resources. In most of the research, the random variables are assumed to be of the same marginal distribution, such as a normal distribution or τ distribution. Thus, the joint distribution of random variables is a multivariate normal distribution or multivariate τ distribution. This concept does not fit the features of the network resource sequence, which has the characteristics of sharp peaks and fat tails. Normal distribution cannot depict asymmetric dependent structures and the tail dependence structure, such as flow, CPU resources, and memory resources. τ distribution can also depict the symmetrical tail structure. None of them can accurately reflect the real situation. In this context, we select the copula functions as the main tools for the correlation analysis. They can put the marginal distributions together to form a joint distribution.

Recently, the use of the copula method has increased because of its ability to model the dependence structure between variables. Copula functions in the research on the correlation of network resources have obvious advantages in the real world. Firstly, there is no restriction on marginal distributions for the use of copula functions. Even marginal distributions may be of different types, which allows us to flexibly construct multivariate distributions. The second advantage of the copula functions is that they can be considered separately from marginal distributions. It describes the flow characteristics of time series by the marginal distribution and characterizes the dependency structure by the copula functions for resource-related structure research. Finally, the copula functions have many structural forms, such as symmetric structure, asymmetric structure, and a complex mix of copula functions. A non-linear asymmetrical positive or negative correlation between flow and resources can be accurately characterized through mixed copula functions. Most of the information is contained in the marginal distribution of each variable, which will not be distorted after connection. Therefore, we use a mixed copula model for modeling the correlations between resources.

Copula theory was put forward by Sklar [30]. Sklar proved that any multivariate distribution function can be decomposed into n marginal distribution functions and a copula connecting function. The converse is also true. Marginal distribution describes the distribution of the variables, while the copula connection function describes the correlation between variables. That is to say that the copula is a function which joins or “couples” a multivariate distribution function (i.e., the distribution of two or more random variables) to its one-dimensional marginal distribution functions. This gives freedom in choosing the univariate marginal distributions once the desired dependence framework has been selected. In turn, this makes it easier to formulate multivariate models.

Definition 1: A bivariate copula connection function refers to the function with the following properties:

(1) The range of *C* is the unit interval [0,1];
(2) (u,v)∈[0,1]×[0,1], where at least one coordinate is equal to zero;
(3) *C* is 2-increasing in the sense that for every *a* ≤ *b* in the measure $\Delta C_{a}^{b}$ assigned by *C* to the 2-box $\left[a,b]=[a_{1},b_{1}]\times [a_{2},b_{2}\right]$ is non-negative; i.e.:
  (1) $$\Delta C_{a}^{b}=\sum_{\left(\varepsilon_{1},\varepsilon_{2})\in \{0,1\right\}}{\left(-1\right)}^{\varepsilon_{1}+\varepsilon_{2}}C(\varepsilon_{1}a_{1}+(1-\varepsilon_{1})b_{1}+\varepsilon_{2}a_{2}+(1-\varepsilon_{2})b_{2})$$

If H(*x*, *y*) is a bivariate distribution function of two correlated random variables of *X* and *Y* with respective marginal cumulative distributions *F*(*x*) and *F*(*y*), a copula *C* can be defined as:

(2) H(x,y)=C(F(x),G(y))

If *F* and *G* are continuous functions, then *C* is unique; conversely, if *C* is a 2-copula and *F*, *G* are distribution functions, then the function given by Equation (2) is a 2-dimensional distribution function. Under the assumption that the marginal distributions are continuous with probability density functions, the joint probability density function then becomes:

(3) h(x,y)=c(f(x),g(y))×f(x)×g(y)

where c is the density function of C; it is defined as:

(4) $$c(f,g)=\frac{\partial^{2}C(f,g)}{\partial f\partial g}$$

By Sklar’s theorem, if the multivariate joint distribution is unknown, then we can construct the joint distribution function through the marginal distribution function and a copula connection function. Conversely, using the inverse function of the marginal distribution function and the joint distribution functions, we can deduce the corresponding copula connection function.

Copula functions are commonly divided into three categories: n-dimensional normal copula, t-copula, and Archimedean copula. The n-dimension normal copula distribution function and density function are as follows, respectively:

(5) $$C(u_{1},u_{2},...,u_{N};\rho )=\Phi_{\rho }(\Phi^{-1}(u_{1}),\Phi^{-1}(u_{2}),...,\Phi^{-1}(u_{N}))$$

(6) $$c(u_{1},u_{2},...,u_{N},\rho )=\frac{\partial^{N}C(u_{1},u_{2},...,u_{N},\rho )}{\partial u_{1}\partial u_{2}...\partial u_{N}}={\left|\rho \right|}^{-\frac{1}{2}}\exp(-\frac{1}{2}\xi^{\prime }(\rho^{-1}-I)\xi )$$

where ρ is an N-symmetric positive definite matrix of which the diagonal elements are all 1, |ρ| represents a square determinant, $\Phi_{\rho }$ indicates an N-element standard normal distribution whose correlation coefficient is ρ and its edge distribution is a standard normal function, $\Phi^{-1}$ represents an inverse function of the standard normal distribution function $\xi^{\prime }=(\Phi^{-1}(u_{1}),\Phi^{-1}(u_{2}),...,\Phi^{-1}(u_{N}))$, and I is an identity matrix. For the binary case, the linear correlation coefficient between variables is set to ρ, and the bivariate normal copula can be expressed as follows:

(7) $$C^{Ga}(u,v;\rho )=\int_{-\infty }^{\Phi^{-1}(u)}\int_{-\infty }^{\Phi^{-1}(v)}\frac{1}{2\pi \sqrt{1-\rho^{2}}}\exp\left\{-\frac{s^{2}-2\rho st+t^{2}}{2(1-\rho^{2})}\right\}dsdt$$

The multivariate distribution function and density function of t-copula expressions are, respectively, as follows.

(8) $$C(u_{1},u_{2},...,u_{n};\rho ,k)=t_{\rho ,k}(t_{k}^{-1}(u_{1}),t_{k}^{-1}(u_{2}),...,t_{k}^{-1}(u_{N}))$$

(9) $$c(u_{1},u_{2},...,u_{n};\rho ,k)={\left|\rho \right|}^{-\frac{1}{2}}\frac{\Gamma (\frac{k+N}{2}){\left[\Gamma (\frac{k}{2})\right]}^{N-1}}{{\left[\Gamma (\frac{k+1}{2})\right]}^{N}}\frac{{\left(1+\frac{1}{k}\xi^{\prime }\rho^{-1}\xi \right)}^{-\frac{k+N}{2}}}{\prod_{i=1}^{N}{\left(1+\frac{\xi_{i}^{2}}{k}\right)}^{-\frac{k+1}{2}}}$$

where parameters ρ and |ρ| are same as Equation (6), $t_{\rho ,k}$ indicates a t-distribution whose correlation coefficient is ρ and its freedom is *k*, $t_{k}^{-1}$ represents an inverse function of the t- distribution function $\xi^{\prime }=(t_{k}^{-1}(u_{1}),t_{k}^{-1}(u_{2}),...,t_{k}^{-1}(u_{N}))$. For the binary case, the linear correlation coefficient between variables is set ρ, t-copula with k degrees of freedom can be expressed as follows:

(10) $$C^{t}(u,v;\rho ,k)=\int_{-\infty }^{t_{k}^{-1}(u)}\int_{-\infty }^{t_{k}^{-1}(v)}\frac{1}{2\pi \sqrt{1-\rho^{2}}}{\left[1+\frac{s^{2}-2\rho st+t^{2}}{k(1-\rho^{2})}\right]}^{-\frac{k+2}{2}}dsdt$$

Genest and Mackay [31] gave the definition of Archimedean copula distribution function:

(11) $$C(u_{1},u_{2},...,u_{N})=\{\begin{array}{cc} \varphi^{-1}(\varphi (u_{1}),\varphi (u_{2}),...,\varphi (u_{N})), & \sum_{i=1}^{N}\varphi (u_{i})\leq \varphi (0)\\ 0\;, & \sum_{i=1}^{N}\varphi (u_{i})>\varphi (0) \end{array}$$

where the function φ(u) is called Archimedean copula function generator, for u∈[0,1], $\varphi^{\prime }(u)<0,\varphi^{\prime \prime }(u)>0$, it means that φ(u) is a convex decreasing function. $\varphi^{-1}(u)$ is the inverse function of φ(u), and is a continuous and monotonously non-increasing function on the interval [0,+∞). The Archimedean copula function is uniquely determined by generators.

Excellent and detailed discussions on copulas can be found in [32]. Most of the studies indicated that several families of Archimedean copulas—including Frank, Clayton, and Gumbel—have been popular choices for dependence models because of their simplicity and generation properties [33]. Different families are necessarily needed for different marginal distributions. The normal-copula, Frank-copula, and tau-copula fit the distribution of a symmetric tail. They are unable to capture correlation relationships between the random variables with asymmetric tails. The tail correlation coefficient of bivariate normal-copula and Frank-copula is 0. In the tail of the distribution, variables are independent of each other. Bivariate Gumbel-copula and Clayton-copula have an asymmetric fat tail. They can capture asymmetric relationships between variables. The Gumbel-copula implies a higher dependence in right tails of the marginal distributions. The Frank-copula implies asymptotic dependence in right tails.

The choice of the copula model is based on the previous analysis, and we have chosen to employ Gaussian, Student’s *t*, Gumbel, and Clayton copulas. If only one parameter appears in a copula function, this parameter usually reflects the strength of the dependence.

The bivariate Clayton-copula function is defined as follows, where is an association parameter, and *u* and *v* are the relevant variables.

(12) $$C(u,v)=\max({\left[u^{-\alpha }+v^{-\alpha }\right]}^{-\frac{1}{\alpha }},0)\;\alpha \in [-1,+\infty )$$

The bivariate Frank-copula is defined as:

(13) $$C(u,v)=-\frac{1}{\alpha }\ln(1+\frac{\left(e^{-\alpha u}-1)(e^{-\alpha v}-1\right)}{e^{-\alpha }-1})\;\alpha \in (-\infty ,+\infty )$$

The bivariate Gumbel-copula is defined as:

(14) $$C(u,v)=\exp(-{\left[{\left(-\log u\right)}^{\alpha }+{\left(-\log v\right)}^{\alpha }\right]}^{\frac{1}{\alpha }})\;\alpha \in [1,+\infty )$$

where the three copula are defined with u,v(u,v∈(−∞,∞)\{0}).

We will assume that marginal distributions are of the form while considering copula-based models for multivariate time series.

It could be deduced by common statistical methods (e.g., skewness and kurtosis) that time series of flow, CPU, memory, and resources sequences are fat-tail, non-linear, non-normal, and time-varying. It is not depicted by the known distribution because there are no relevant models to describe the overall distribution when there is a large fluctuation. Therefore, nonparametric density estimation methods have been used. The model employs the kernel smoothing density estimate and empirical distribution estimate algorithms to assess the general distribution.

The empirical distribution is a staircase function with the location of the drops placed randomly. It estimates the cumulative distribution function underlying of the points in the sample, which is an integral of the empirical density distribution. The empirical density distribution is given by:

(15) $$f_{n}(x)=\{\begin{array}{c} \frac{f_{i}}{h_{i}}=\frac{n_{i}}{nh_{i}}\;x\in I_{i},i=1,2,...k\\ 0\;x\notin I_{i} \end{array}$$

where x is on behalf of *n* sample observations. *h*_i_ represents the width of each window, and *n*_i_ is the number of observations falling in each *h*_i_.

In many statistical problems, it is required to estimate the overall probability density distribution from the sample, and the common estimation methods are divided into parametric and non-parametric methods. The parametric method assumes that the overall distribution obeys some known distribution; namely, the density function of the form is known, wherein it needs to estimate parameters from the sample and it relies on hypothesized distribution. Whereas, there is no such trouble in the non-parametric method.

As can be seen from the definition of empirical density function, the estimated value of some point is closely related to the number in the vicinity of the points, if sample points near x are relatively dense, the density estimate function is relatively large, and relatively small, vice versa. Although $f_{n}(x)$ satisfies the rule, its value depends on the size of the division, and the value of $f_{n}(x)$ is a constant on each small interval. To overcome the shortcoming of interval division, we assume that there is a neighborhood where *x* is the center and $\frac{h}{2}$ is the radius. When *x* changes, the neighborhood also changes position. We can use this number of sample points falling within the neighborhood to estimate the density function value at point *x*. Thus, the neighborhood function (Parzen-window function) with a center of x and a radius of $\frac{h}{2}$ is defined as follows:

(16) $$H(u)=\{\begin{array}{cc} 1, & \left|u\right|\leq \frac{1}{2}\\ 0, & rest \end{array}$$

where u is the distance between any point and *x*.

When the first sample point $x_{i}$ falls into the neighborhood with a center of *x* and a radius of $\frac{h}{2}$, $H(\frac{x-x_{i}}{h})=1$; otherwise, $H(\frac{x-x_{i}}{h})=0$. Therefore, the total number of samples falling into this neighborhood is $\sum_{i=1}^{n}H(\frac{x-x_{i}}{h})$. The dot density function estimate at x is:

(17) $${\hat{f}}_{n}(x)=\frac{1}{nh}\sum_{i=1}^{n}H(\frac{x-x_{i}}{h})$$

Since the Parzen window density estimation method considered all points in the neighborhood of *x* to be equal, this means all points of the neighborhood are equally important to ${\hat{f}}_{n}(x)$. This method is not reasonable. The better method is determining their dedication according to their distances from *x* in the neighborhood. Many papers [30,32] have proposed many rational kernel functions, such as uniform, triangle, Gaussian, cosinus, quaritic, and so on.

$X_{i}$ is the sample of the population. At any point *x* in the overall density function, *f*(*x*) of the kernel density estimation is defined as:

(18) $$f_{h(x)}=\frac{1}{nh}\sum_{i=1}^{n}K(\frac{x-X_{i}}{h})$$

where K is called the kernel function, *h* is called the window width. In this model, K uses a Gaussian kernel function to estimate, which is defined as follows:

(19) $$K(u)=\frac{1}{\sqrt{2\pi }}e^{-\frac{1}{2}u^{2}}$$

where u is the estimate of the sample mean.

We fit resource sequences through an overall analysis of the kernel density function and empirical density function. The fitting results of a marginal distribution directly affect the estimation of parameters in the copula connection function. After calculating the marginal of X (U = *F*(x)) and Y(V = *G*(x)), we can choose an appropriate copula connection function from their bivariate histogram.

More complex copula functions usually contain one or more parameters, which are also called association parameters. A single parameter in a copula function usually reflects the strength of the dependence. Similarly, there are unknown parameters in the marginal distribution function. We must estimate both types of parameters using the observed samples. Inference Functions for Margins (IFM) and Canonical Maximum Likelihood (CML) methods are adopted in our method.

The method of prediction by copulas is as follows:

1. Calculate the marginal distribution of two variables (flow and memory are chosen in our simulation).
2. Select the appropriate copula function to describe the structure of the random variables according to the density function.
3. Estimated copulas connect for unknown shape parameters in the model.
4. Construct a joint distribution function of independent and dependent variables using a copula function, and then analyze the relevance of the independent variable, dependent variable, and the relevant model. We perform a meticulous study of sample values of the unknown dependent variable probability distribution and the relationship between the joint distributions on the basis of combining with the independent variable probability distribution characteristics of known sample values. Therefore, we can predict the unknown value of the dependent variable.
5. At time t + 1, variable X_t+1_ can be measured or estimated under the condition of X_t_. through the copula connection function and joint distribution, combined with the polynomial fitting of the variable Y_t_, Y_t+1_ can be predicted according to the relation between the variables.

This gives the possibility of calculating the probability of the occurrence of dangerous super-critical events. Different families are necessarily needed for different marginal distributions. It should be mentioned that the copula is assumed to be chosen by the designer based on their experiences. The most commonly used copulas are the Gaussian copula for linear correlation, Gumbel copula for extreme distributions, and the Archimedean copula and the t-copula for dependence in the tail.

On the sample time scale, we can choose a relatively long period of time to build a joint distribution function. The two-dimensional curved surface reflects the combination of the various related resources and higher frequency numbers. The more sample statistics there are, the more noticeable the long-term effects of the results are. The polynomial fitting is a reaction of a correlation of short-term behavior. We can predict a dependent variable through the combination of long-term and short-term behavior. In the case of multiple combinations of different copulas, we choose the maximum combination as the result of the prediction. The end results can effectively estimate the range of resource changes, and provide important references for subsequent safety.

#### 3.1.2. Risk Assessment

In risk prediction analysis of network resources, it is quite rare that resource starvation occurs at the same time. It is usually the case that some resource is firstly exhausted, but there are still some links between the resources. It is not enough to only know the marginal distribution of a single network performance index; the dependency structure between the indexes also needs to be analyzed. Prediction of an index alone may ignore the influence of other factors, resulting in mistakes. Although the n-copulas connection function can describe the dependence structure among the variables, its high dimension leads to difficulty in calculation. We need to decompose a pair of multivariate joint probability density functions into several low-dimensional functions.

Set an n-dimensional random vector $X=\left(X_{1},X_{2},\cdots ,X_{n}\right)$; its joint distribution function is $F\left(x_{1},x_{2},\cdots ,x_{n}\right)$. According to the Sklar theorem, the multivariate distribution function can be represented by a copula connection function and marginal distribution function of random variables, as follows:

(20) $$F\left(x_{1},x_{2},\cdots ,x_{n}\right)=C\left(F_{1}\left(x_{1}\right),F_{2}\left(x_{2}\right),\cdots ,F_{n}\left(x_{n}\right)\right)$$

**Figure 3:**
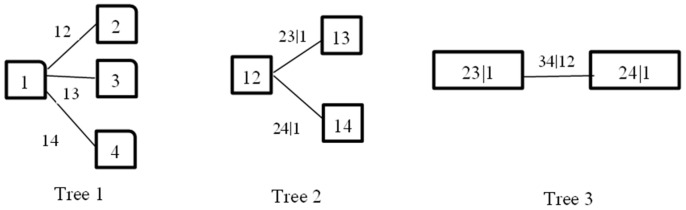
Frame of link-transmitting topology control.

If the joint distribution function and the marginal distribution function are given, the multivariate joint density function can be expressed as:

(21) $$f\left(x_{1},x_{2},\cdots x_{n}\right)=c_{12\cdots n}\left(F_{1}\left(x_{1}\right),F_{2}\left(X_{2}\right),\cdots ,F_{n}\left(x_{n}\right)\right)\cdot f_{1}\left(x_{1}\right)\cdot \cdots \cdot f_{n}\left(x_{n}\right)$$

where $c_{12\cdots n}\left(.\right)$ represents an n-dimensional copula density function and $f_{i}\left(x_{i}\right)$ represents an edge density function. The edge density function is relatively easy to estimate, but the inter-dependent variable multidimensional description of the structure is relatively complicated. Based on the two binary copula diversity of choice, we can decompose the n-dimensional copula density function into a product of several pair copula density functions in order for it to be more convenient to describe the complex multiple dependent structures. In practice, each pair of copula functions may use different types so that the data is better fitted. For high-dimensional copula density functions, pair copula decomposition has several logical structures. Reinhold [34] introduced the graphical C-vine to describe the logical structure. C-vine is the most widely used visualization in describing the logical structure; the structure is shown in Figure 3.

C-Vine consists of trees, nodes, and edges. Figure 3 depicts a logical decomposition structure of a four-dimensional C-Vine. In Figure 3, there are j (j = 1, 2, 3) trees. Each tree, respectively, has a total of (5 − j) nodes and (4 − j) edges, each edge corresponds to a pair copula connected density function, and edge labels correspond to subscripts of the pair of copula density functions; for example, “23|1” corresponds to copula connection density function $c_{23|1}(*)$. According to the logical structure of the C-Vine, an n-dimensional copula connection density function $C\left(F_{1}\left(x_{1}\right),F_{2}\left(x_{2}\right),\cdots ,F_{n}\left(x_{n}\right)\right)$ can be decomposed into the following form:

(22) $$C\left(F_{1}\left(x_{1}\right),F_{2}\left(x_{2}\right),\cdots ,F_{n}\left(x_{n}\right)\right)=\prod_{j=1}^{n-1}\prod_{i=1}^{n-j}c_{j,j+i|1,2,\cdots j-1}\left(F\left(x_{j}|x_{1},\cdots x_{j-1}\right)\right)\cdot F\left(x_{j+i}|x_{1},\cdots ,x_{j-1}\right)$$

Among them, each pair copula connection density function contains a pair of conditional distribution functions. Equation (23) can be given by Reference [34]:

(23) $$F\left(x|\nu \right)=\frac{\partial C_{xv_{j}|v_{-j}}\left(F\left(x|v_{-j}\right),F\left(v_{j}|v_{-j}\right)\right)}{\partial F\left(v_{j}|v_{-j}\right)}$$

where $v_{j}$ represents the first *j* elements of vector V, $v_{-j}$ on behalf of removing the *j* elements from the vector V. When vector V contains only one element, Equation (23) can be converted to Equation (24).

(24) $$F\left(x|v\right)=\frac{\partial C_{xv}\left(F\left(x\right),F\left(v\right)\right)}{\partial F\left(v\right)}$$

Through the definition of the C-Vine and Figure 3, we can see that the C-Vine is suitable for describing a dataset with guide variables. Network resources include traffic, CPU, and memory, and the traffic into the node has a certain impact on other resources, which can be regarded as a guide-variable. The rest of the variables are called affected-variables, and C-vine is used to describe the correlation and predict whether the node is facing an excessive consumption of resources or risk. Calculation of C-Vine requires that the edge distributions are provided in advance [35], and we can use the Generalized AutoRegressive Conditional Heteroskedasticity (GARCH) model to fit them.

The time series of network resources has an obvious fluctuation. The data curve exhibits a characteristic of peak and fat-tail. The GARCH model can better describe the fluctuation of data, while the t-distribution can describe the peak and fat-tail. Therefore, the GARCH-t model is employed to calculate the marginal distribution of each index.

First, the GARCH-t (1,1) model is used to estimate the marginal distribution of a single index ($F\left(x_{i}\right)$). Assuming there are n network resources, $\{x_{1},x_{2},\cdots ,x_{n}\}$ is a set of observed values of one resource, considering the following GARCH-t (1,1) model:

(25) $$\left\{ \begin{array}{l} x_{t}=c_{0}+c_{1}x_{t-1}+a_{t}\\ a_{t}=\sigma_{t}\xi_{t}\\ \sigma_{t}=\omega +\alpha a_{t-1}^{2}+\beta \sigma_{t-1}+\gamma e_{t-1}^{2}L\\ \xi_{t}\sim SkT(d,\lambda ) \end{array}\right.$$

where mathematical symbols $\left(w,\alpha ,\beta ,\gamma ,c_{0},c_{1},d\right)$ are the model parameters, $x_{t}$ is a time series, $a_{t}$ are used to reflect the fluctuation of the time sequence. Since t-distribution can capture the characteristics of a peak and fat-tail well, $\xi_{t}$ is set as a t-distribution with the degree of freedom d. L is an indicator function. The conditional distribution of $x_{t}$ in the t + 1 time ($F\left(x_{t+1}\right)$) can be expressed as the form $P\left(X_{t+1}\leq x\right)$, which can be derived through Equation (20).

(26) $$\begin{array}{l} F\left(x_{t+1}\right)=P\left(a_{t+1}\leq x-\mu \right)=P\left(\xi_{t+1}\leq \frac{x_{t}-c_{0}-c_{1}x_{t-1}}{\omega +\alpha a_{t-1}^{2}+\beta \sigma_{t-1}+\gamma e_{t-1}^{2}L}\right)\\ =t_{d}\left(\frac{x_{t}-c_{0}-c_{1}x_{t-1}}{\omega +\alpha a_{t-1}^{2}+\beta \sigma_{t-1}+\gamma e_{t-1}^{2}L}\right) \end{array}$$

where $t_{d}\left(.\right)$ is a t-distribution function with the degree of freedom d. Through the maximum likelihood estimation, the GARCH (1,1) model parameters are obtained from the sample $\{x_{1},x_{2},\cdots ,x_{n}\}$, Equation (21) can present the marginal distribution function at time t + 1, then the marginal density function is obtained by taking the derivative of Equation (21).

As mentioned before, we selected network traffic, device CPU, and memory as the cyber resource index; three time series were obtained by the resource monitor, and sampling points were formed by sampling. The GARCH model is used to obtain the marginal distribution of each index, and then select the appropriate copula type.

The calculation procedure of the joint density function is stated as follows:

1. The time series of a certain index ($x_{i}=\{x_{i,1},x_{i,2},\cdots ,x_{i,n}\};i=1,2,3$) is selected, and—combining with the marginal distribution function $F\left(x_{i}\right)$—we can estimate the parameters of the copula pair density function on the first tree, such as $c_{12}\left(F_{1}\left(x_{1}\right),F_{2}\left(x_{2}\right)\right)$, $c_{13}\left(F_{1}\left(x_{1}\right),F_{3}\left(x_{3}\right)\right)$, etc. According to Equation (3) and the parameters of the copula pair function in Tree 1, the conditional distribution function of Tree 2 is obtained, and the corresponding observed values can also be calculated.
2. Under a certain confidence level, the distribution function of the normal rate of the index combination within the time t is defined as:
  (27) $$P\left(X_{t}\leq VaR\left(\alpha \right)\right)=\alpha$$
  It is expressed at a given confidence level. In the next period of time t, the risk rate will not exceed some value rate (VaR).
3. If there are n resources, $X_{i,t}(i=1,2,\cdots ,n)$ is the normal value of resource. VaR is defined as Equation (23). $X_{t}=\max(X_{1},X_{2},X_{3})$ is the highest value of n resources at each time. After the introduction of copula pair decomposition, Equation (7) can be written in the form of the integral:
  (28) $$\begin{array}{l} P\left(X_{t}\leq VaR\left(\alpha \right)\right)=P\left(\max(X_{1},X_{2},X_{3})\leq VaR\left(\alpha \right)\right)\\ =\int \cdots \underset{\max(X_{1},X_{2},X_{3})\leq VaR\left(\alpha \right)}{∭}f\left(x_{1},x_{2},\cdots ,x_{n}\right)dx_{1}\cdots dx_{n}=\alpha \end{array}$$

### 3.2. Active Defense of Incomplete Strategy of a Minority Game

#### 3.2.1. The Basics of the Minority Game Model

In the recent past, properties of the minority game (MG) model were explored exhaustively. Basically, this model is designed for the El-Farol problem, which contains an El-Farol Bar in Santa Fe where Irish music is proposed every Thursday night. It is supposed that a limited population of consumers (100 agents) have to decide to go to the bar every weekend. However, if there are more than 60 people in the bar at the same time, the bar will become very crowded. It is expected that the consumption value of all those consumers who cannot enjoy life in a crowded bar is less than zero. Therefore, it is an iterated pure coordination game. There is a common rule of the game that dominates the choice of the individual: go to the bar or stay at home. The main difficulty of the problem that arises due to non-interpersonal communication is that the players have to infer from the past the number of people that will decide to go to the bar on the next Thursday. In this particular situation, no model exists for a deductive decision by the players. The players are condemned to use inductive reasoning. From numerical simulation, it is found that the average number of people who go to the pub always converges to a certain value, called the “Attractor”. This parameter is also known as a tolerance factor, as it determines the range of accepted values for a normal state of the network.

Challet assumes that there are N participants for several rounds (time t indicates that the game is in the first t round) [36]. In each round, individual participants predict results according to their respective strategy, and make a choice between 0 and 1 in the room. After several rounds of the game, a binary sequence is formed, called the history of the game.

In 2007, Sun proposed a new model of the incomplete minority game (IMG), which features a default hierarchy of the rules [37]. In this model, strategies may be incomplete. Random bits can be introduced into the strategies of agents so that the secondary rule can be applied in the absence of the primary one. When all strategies cannot predict cases, the agent takes random action in the game. This model improves the overall performance by one order of magnitude with less memory steps and a more stable combination of strategies.

#### 3.2.2. Improved Minority Game Model

In this section, we proceed to describe the MG model for the proposed approach after obtaining the timing of active defense initiation.

Recently, we proposed an improved model called incomplete strategy minority game with a preconfigured attractor [38]. According to this model, the minority game is an appropriate measure for describing the competition systems.

From the viewpoint of resource utilization, resource collapse is the result of multiple network traffic behaviors competing for limited resources, such as memory, bandwidth, etc. Some real-time resource information of the node (e.g., the resource utilization ratio and its rate of change) is readily available from the resource manager of the server, which is usually a built-in tool. The name of the command is “perfmon.exe” under the Windows platform, and that of “sar” under Linux. At deployment time, all we add is a data collection module instead of a special monitoring module. This means that each node is equipped with a sensor to deliver the real-time resource information to the gateway, which we call virtual sensors. The information will be collected at the early alert module for statistical analysis. We do not allow the competition to continue to the worst scenario for a resource collapse predicted by the copula model, but try to suppress or digest the threats. To simplify the model, we establish the model using bandwidth resources as the suffering of the attacks. Other resources are also treated similarly. Overall targets of the network should involve the best strategy that makes full utilization of resources and helps the participants to maintain normal performance and efficient resource utilization. 

In this game model, there is a central node called the firewall, and various participants of flow behavior. The firewall is responsible for the coordination of the behavior of various participants, but is unable to determine the class of flow (normal behavior, aggression). Thus, the firewall must simulate the choice and strategies of each participant.

The history of resource state and choice information affect the decision of the participants. In the process of the game, each class of flow behavior can maintain memory with a length of m. The memory information is composed of the last two resource states and the choice of each flow behavior.

It is assumed that the maximum number of the flows into the network (or the node) is n. However, the network (or the node) can only work normally in a maximum normal scope of b number of flows. Let the real-time number of flows be x. When x > *b*, the network (or the node) will collapse for lack of resources. However, the attractor’s value is preconfigured according to the performance of the network (or the node). The value of the attractor should match the dynamics of the network (or the node). Hence, for a particular network environment, a careful investigation of performance should be performed, which usually depends upon hardware performance and requirements of the service.

The proposed work provides a modified IMG with the attractor. It is carried out in the following steps: 

Step 1. Each packet through a firewall is classified into categories by the four-tuple representation {*source-address*, *destination-address*, *port-number*, *protocol*} as a basic division. The destination address is the node that needs to be protected in the network, and the source address is arbitrary. The categories with the same port and destination address can be merged into one category {*flow_n_*, *dest-addr_n_*, *port_n_*}.

Step 2. The firewall establishes a standard table of incomplete strategies for flow behavior (Table 1) corresponding to the memory information with a length of 4. The decision of each game participant is affected by their two previous resource states and the choice of each flow behavior. Assumption 1: suppose the firewall only maintains memory with a length of m for each flow behavior, where m is consistent with the flow’s memory space. Resource state is measured after determining the strategies. The history information (including the resource state and choice) of each flow form a 0–1 sequence {*strategy_n−2_*, *strategy_n−1_*, *state_n−2_*, *state_n−1_*, *choice_n_*}.

The choice of the current flow depends on two factors: resource state and choice. The choice (or strategy) of each flow behavior can be divided into two kinds, depending upon circumstances: send (1) or destroy (0). Resource state is also divided into two kinds: tension (1) or loose (0). We can form a table to predict the next action of a flow based upon thorough analysis of the logical relationship of the four parameters. Obviously, more historical data leads to better accuracy predictions. This model uses only the last two historical strategies due to the limited memory space and the real-time requirements. Therefore, the strategies of the flows are not complete and reliable. It cannot give an effective forecast based upon historical information of flows, but shows a relatively trend prediction. The firewall acts as an agent of each flow, and chooses the strategy for an alarm signal received from the copula model. For example, {0,0,1,0,0} shows that the flow adopts the strategy of not sending in the last two steps, and the last state is 1, which means a shortage of resources. We have reason to believe that the flow is of the attack traffic class and should not to be sent in the current strategy. If it is non-attack traffic, it would be sent at least one time in the last two steps. {1,0,1,0,0} shows that the flow has been sent at the second step from the bottom, but the resource state became 1, so the flow can be suspected that it made a devotion to the shortage of resources. The current strategy should be adopted for not sending it. To quickly reduce traffic, set the number of sending strategies to less than half of the total number of strategies. 

Forwarding strategy proportion is defined as α, it represents a packet ratio in which a packet can be selected to transmit in each round. The condition α<0.5 ensures that the result of the game must be elected a minority in every round. When we want to reduce the flows more quickly, α can be set smaller, correspondingly, as the normal behavior of the traffic will also be filtered less. In the simulation, we set α to a value of $\frac{7}{16}$ to increase legitimate packets of penetration rather than quickly reduce the traffic. The impact of α will be discussed in Section 4.2.

Step 3. Read the current resource status of the protected node, update each flow sequence {*strategy_n−2_*, *strategy_n−1_*, *state_n−2_*, *state_n−1_*, *choice_n_*}, move *state_n−1_* forward as *state_n−2_*, and fill the current state as *state_n−1_*.

Step 4. Each flow chooses one out of the 16 strategies mentioned in Table 1. It sets *history_num_flag* (on behalf of the flow that is about to be sent) to 1 based upon its best working strategy in each turn.

Step 5. Modify each flow’s sequence of strategies. Move *strategy_n−1_* forward as *strategy_n−2_*, and update the current strategy as *strategy_n−1_*.

Step 6. Sort the number of flow behaviors. Suppose the result is a sequence called nf {*num_flow_1_ < num_flow_2_ < …… < num_flow_m_*}; calculate the minimum value of *k* to satisfy the condition (*num_flow_1_ + num_flow_2_ + …num_flow_k_*) > *attractor*. Set *minor_num_flag* of all flows in the scope of *k* to 1.

Step 7. Count the number of the flows whose history_num_flag is equal to 1 as *sum_history*. If *sum_history* < *attractor*, then go to Step 8, and add the flows who meet *history_num_flag* = 0 and *minor_num_flag* = 1 by ascending n flows sequence. Set for these flows’ history_num_flag = 1 until sum_history = attractor. If *sum_history* > *attractor*, decrease the flows by descending order of *n* sequence and set flows’ *history_num_flag* = 0. This process continues until *sum_history* = *attractor*.

Step 8. Let all the flows where *choice_n_* = 1 pass through a firewall, and destroy all of the flows where *choice_n_* = 0.

In a typical game, each flow determines the action of the current game based on a historical strategy table. If a flow chooses to send, it means that the flow predicts that its sending will not lead the state of resources into more tension. However, a destruction choice of flow indicates that it predicts the state of resources is tension. It prefers destruction to avoid competing for resources. The game will keep sending the minority flows to avoid competing for resources, based on the history of the minority game statistics. The arrival of any new flow behavior into the network will become the minority flow. If some traffic becomes a majority, then the strategy table is updated from 0 to 1, or from 1 to 0 by the concerned flows after all flows make their choice to be sent or destroyed. The firewall server will collect the resource state of the protected node as a feedback factor, and modify the history strategy sequence of flows. This completes a round of the game.

At the beginning, the flows’ strategy of the first two rounds can be set randomly, and the resource state is recorded with the passage of time. As the game progresses, strategies in the table are corrected. In the first round, due to the lack of previous information, each flow prefers to think itself as a minority, so their choices are directly being set to 1, allowing the flows to pass through the firewall, which account for a small proportion in the total number of flows. In the second round, the tense state of the resource indicates that the last choice needs to be improved. Consequently, all of the flows whose choice is 1 are set to 0, the rest are set to 1. If the number of flows with choice *n* = 1 does not reach the attractor, then the firewall repeats Step 8 until it reaches the target. In this process, the normal flow in the second round is likely to be selected to pass through the firewall. In rounds 3 and 4, the flow forms a strategy sequence. Subsequently, more rounds stabilize the strategy table. At the end, the game model makes the network resources tense situation loose, and flow traffic is limited in the scope of an equilibrium state; the resource utilization and fairness of flow become more efficient.

In the incomplete strategy table, the number of entries having *choice* = 1 is slightly lower than that of *choice* = 0, which means that less than 50% of all the flows are prior to be sent. Due to the low proportion of all strategies choosing 1, the result of the selection must be less than 50% of a few behaviors prior to send. Obviously, this reduces the competition for resources, but the resources remain underused, especially in the case of FE. The model goes on increasing the flows which account for a small proportion in the total flows by the attractor. Experimental results show that the number of flows in a node fluctuates in a reasonable range, which approaches closer to the largest efficiency. In particular, we found that the normal flows are able to pass through the firewall at a higher percentage than the method of discarding packets directly. Therefore, this model also achieves the goal of winning.

## 4. Simulation and Analysis

### 4.1. Simulation of the Alert Model

We estimate the marginal distributions first and then estimate the copula function. In order to solve and process the difference of non-same dimension, they must be normalized. Setting the scope of a resource *u* as $\left[u_{\min},u_{\max}\right]$, we use a normalization Equation (29) as follows:

(29) $$q=\frac{u-u_{\min}}{u_{\max}-u_{\min}}$$

where *u* and *q* are the values before and after the conversion, respectively. $u_{\max}$ and $u_{\min}$ are the maximum and the minimum of samples. All of the variables in the following calculations adopt the normalized value of Equation (29).

Firstly, we construct a simulating network in the lab. It is a typical and common scenario: a gateway, internal network, and external network. There are ten attackers on the local network, namely, access video, a news server on a remote network, and a random click on the network video and web pages. We record traffic, CPU, and memory values on the server, along with their corresponding time series.

The example we give is part of server monitoring. In fact, a major reason for the collapse of a server is the overburden on memory and the CPU. Two factors should be monitored at the same time in applications; (flows, memory) and (flows, CPU) are two completely symmetric modules which can function simultaneously. Hence, only one of them is chosen in the experiment.

**Figure 4:**
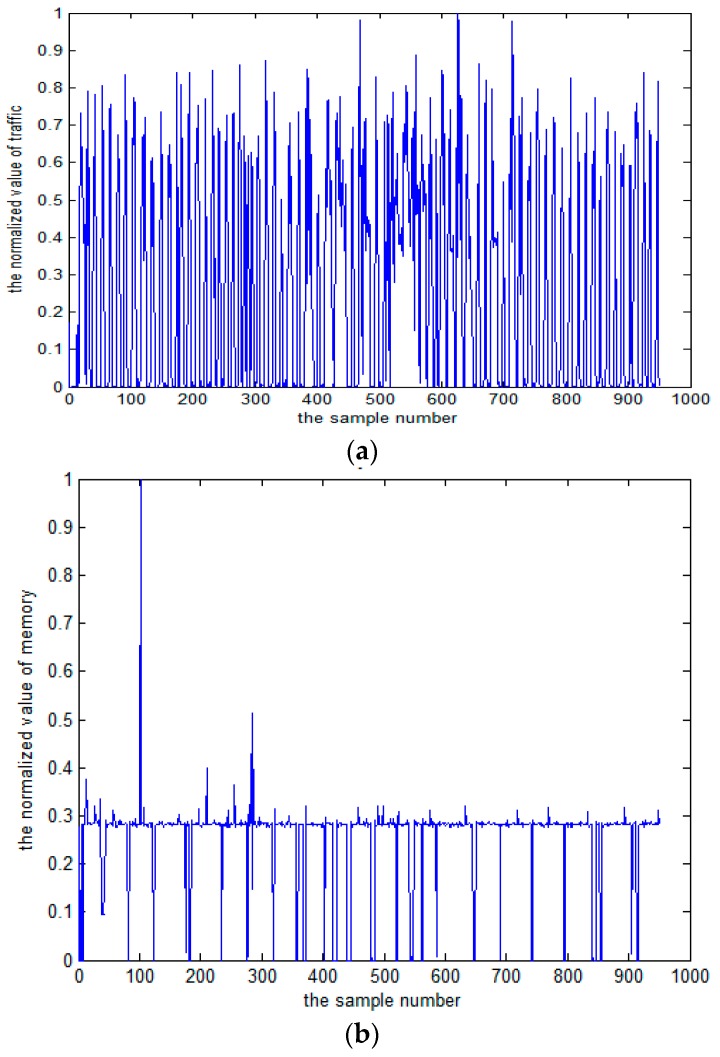
(**a**) Traffic data series; (**b**) Memory data series.

**Figure 5:**
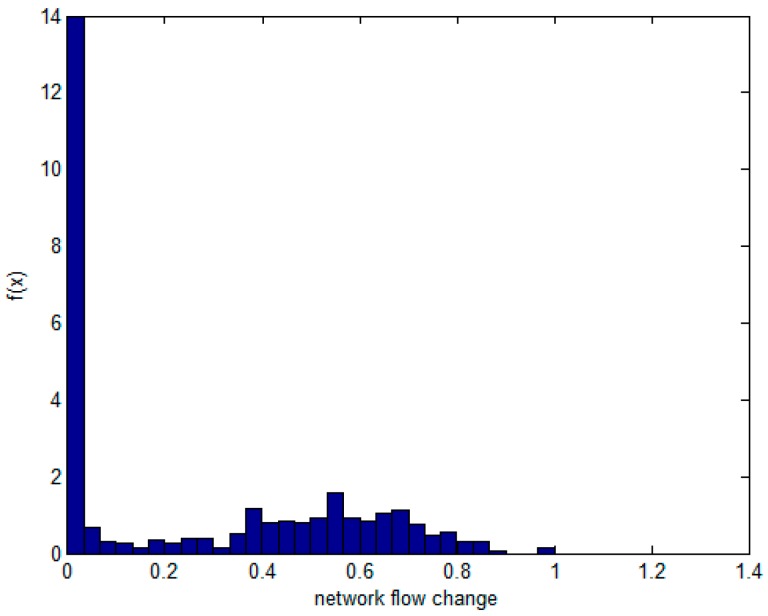
Frequency histogram of flow.

**Figure 6:**
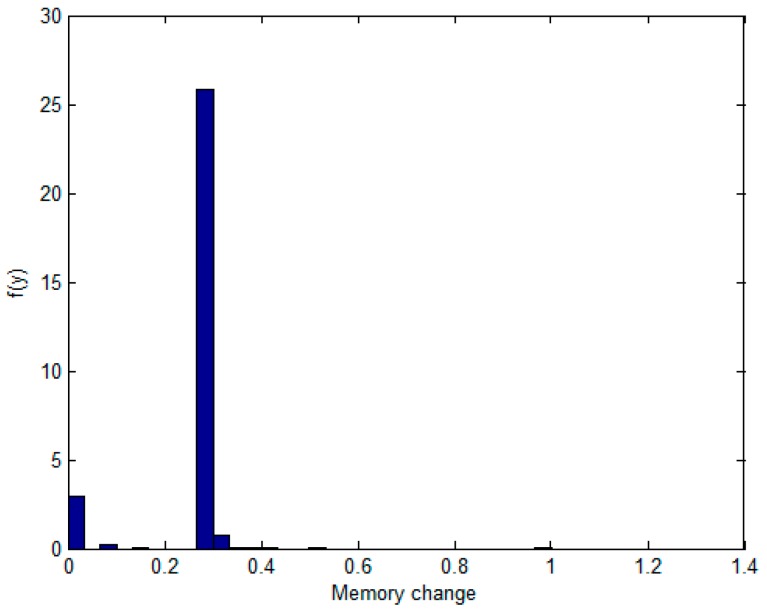
Frequency histogram of memory.

The model is calculated based on the flow variable X and the memory variable Y. According to the initial sample set, the marginal distribution functions of X and Y were determined, respectively. Then, each flow and memory is collected over 1000 sample points (Figure 4). In the last 960 samples, the sample points of the marginal distribution are established. Their frequency histograms are shown in Figure 5 and Figure 6. The rest of the 40 samples are used as test data.

We need to infer the population distribution from the observed data. First, skewness and kurtosis of the sample distribution of the two variables are computed. Both of the variables can indicate the peak and fat tail of the distribution. Skewness is used to indicate whether the frequency distribution of statistical data is symmetrical, and kurtosis is used to reflect the top sharpness or flatness degree of the frequency distribution curve. The normal distribution is one type of light distribution. After calculating X, Y of kurtosis and skewness coefficients from Figure 5 and Figure 6 (*skewness* − *flow* = 0.4716, *skewness* − *memory* = −1.6195, *kurtosis* − *flow* = 1.6640, *kurtosis* − *memory* = 11.9190), we can preliminarily conclude that the two variables are not normally distributed. Then, the Kolmogorov–Smirnov test is used to check if they follow normal distribution, cumulative distribution, exponential distribution, or extremum distribution, and so on. At this point, some MATLAB test functions can be called for normality. From the values of parameters h = 1, and *p* < 0.01, it can be concluded that Y and X do not obey normal distribution. They are parameters in the Kolmogorov–Smirnov testing method which can be used to check whether any sample distribution can be fitted using the usual normal distribution. Moreover, the scatter of the data does not fit any known family of joint distribution, and it may be difficult to specify the joint distribution. Thus, following are details about the major steps of the simulation.

**Figure 7:**
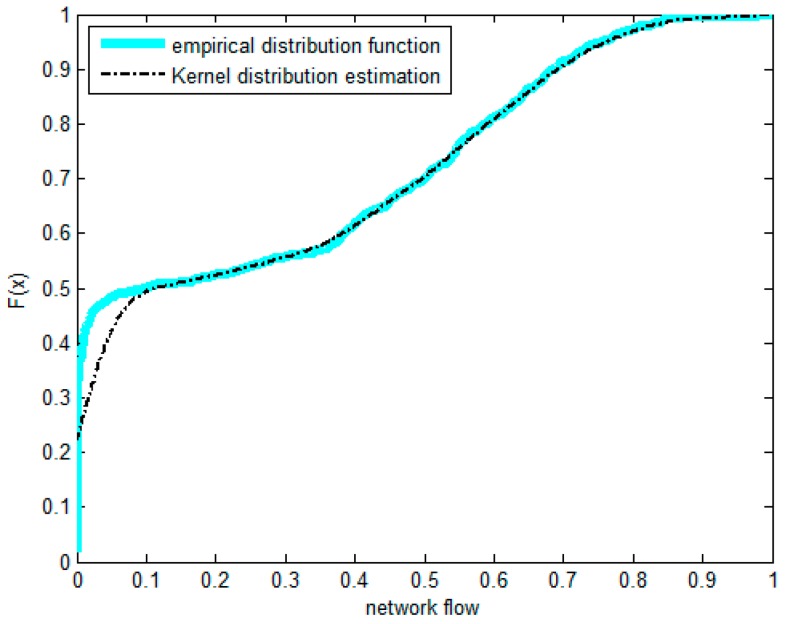
Density function of flow.

**Figure 8:**
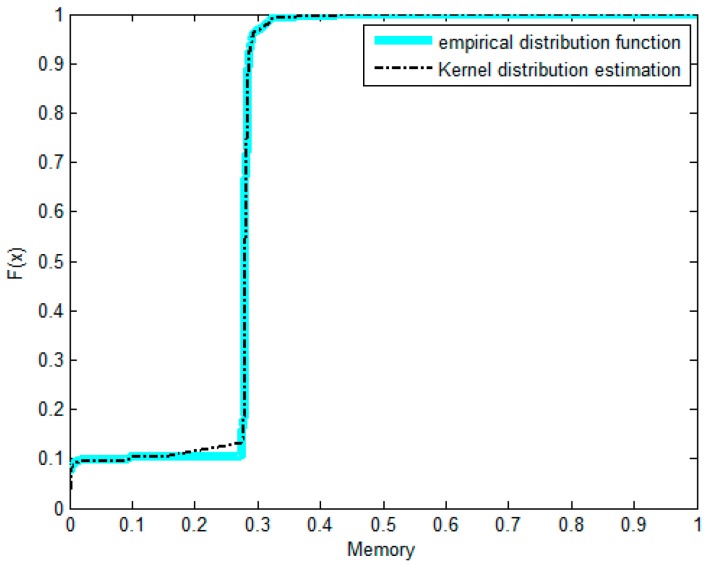
Density function of memory.

**Figure 9:**
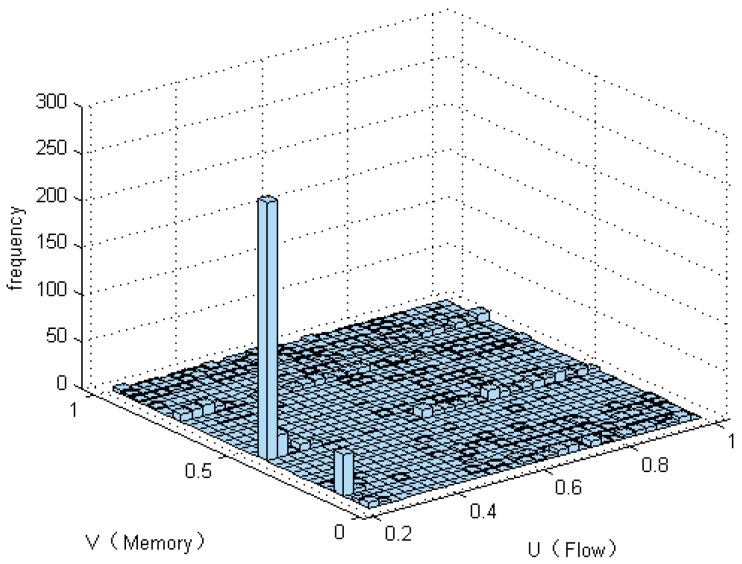
Bi-histogram of memory and flow.

**Figure 10:**
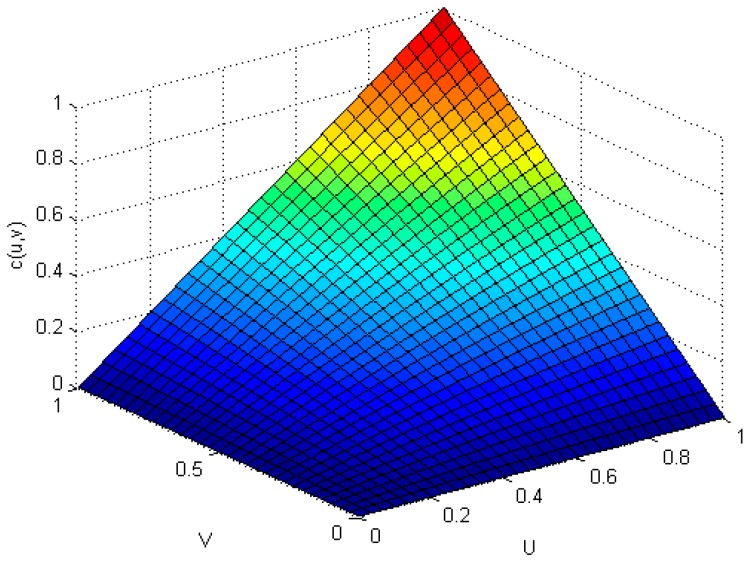
Distribution function diagram of *U* and *V*.

1. The non-parametric methods are used to determine the X and Y of the distribution, including the empirical density function and kernel density function.The polynomial spline interpolation method is used to obtain a continuous curve for the empirical distribution of the original sample. As shown in Figure 7 and Figure 8, the two variables’ empirical distribution function and kernel estimation are basically coincident.
2. The marginal distributions *F*(*X*) and *G*(*Y*) curves are obtained using the above-cited method. A optimal copula connection function can be selected according to the binary (*Ui, Vi*) histogram (Figure 9 and Figure 10), and copula parameters can be estimated using MATLAB programming.

As shown in Figure 10, (*U_i_,V_i_*) is more intensive under the tail of the distribution. The distribution has lower tail dependence with density. This shows that the correlations of the two resources in the depressed period are significantly higher than those of the active period. That is, when the traffic is relatively weak, the impact on the memory resource is greater. The copula function of Equation (30) is constructed by using the previous Equation (14), and then corresponding joint density functions are obtained. For example, in the case of the Gumbel-copula function, the estimated value of N was 1.0236.

(30) $$C(u,v)=\exp(-{\left[{\left(-\log(F(x))\right)}^{\alpha }+{\left(-\log(G(y))\right)}^{\alpha }\right]}^{\frac{1}{\alpha }})\;\alpha \in [1,+\infty )$$

3. The sample value of the known variable X in t + 1($x_{t+1}$) is 0.0000978. The corresponding marginal distribution probability is computed through interpolation. Put α into the Gumbel-copula formula.
4. Proceed with the fitting of the samples. We obtain an approximate Equation (31) as follows:
  (31) $$C=a_{1}v^{m}+a_{2}v^{m-1}+...+a_{m}v+a_{m+1}$$

In Equation (31), $\alpha_{i}$(i = 1,2…m) is a polynomial coefficient, and m depends on the fitting situation. This method makes a cubic polynomial curve-fitting for every 50 points. From the other viewpoint, Equation (32) expresses the relationship between the marginal distribution of Y and the joint distribution. These two equations can be combined to calculate the value of $y_{t\;+\;1}^{*}$. The predictive value of Y at time t + 1 is as given below:

(32) $$y_{t+1}^{*}=G^{-1}(y^{*})$$

where $G^{-1}(y^{*})$ is the inverse function of marginal distribution G, and the predictive value of the memory at t + 1 time is 0.2807.

**Figure 11:**
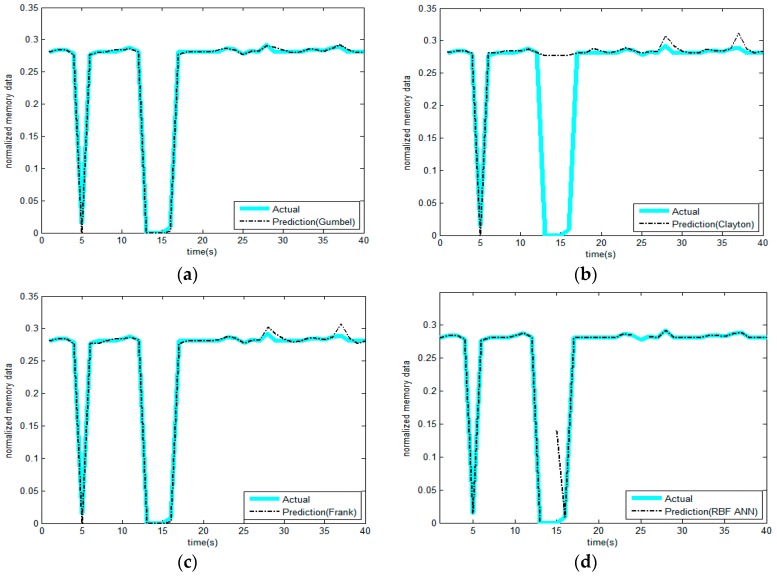
Comparison between predictive value and actual value of memory through four methods. (**a**) Gumbel-copula; (**b**) Clayton-copula; (**c**) Frank-copula; (**d**) RBF-ANN.

5. Repeat the previous step, continue to predict the values following, and we can obtain the results as shown in Figure 11a.

We used three kinds of copulas (Gumbel, Frank, and Clayton) to select the best one among them. Figure 11a–c illustrate the memory prediction results versus actual memory data for each selected copula model. The prediction results are mostly in accordance with actual values. In contrast, Gumbel-copula seems to perform better on the whole. We can estimate the upper memory limit in a better way by using the Frank-copula and Clayton-copula models. Clayton-copulas are not sensitive to the lower memory limit. The lower limit of the resources to be protected is safe and will affect the network. However, protective measures are required for the network or node when the value of resources exceeds the threshold. Therefore, we used the Gumbel-copula model to predict the resources. The rest of the copulas could not manage to capture the dynamic upper information.

As discussed in Section 3.1, the same data is predicted using Radial basis function (RBF), and the prediction results are shown in Figure 11d. It can be seen from the figure that, for the non-linear data that features peaks and fat tails, this method cannot produce effective results, and some of the prediction results are jump outliers. However, the results from the copula function are very robust.

In order to further test the performance of the proposed model, we used R-square (R^2^) and root mean squared error (RMSE) to depict the difference between them. The “deterministic coefficient” can measure the quality of fitting using the variation of data. Its normal value is in the range [0, 1]. The closer to 1 it is, the greater the ability of the variable in Equation (33) to illustrate the original function and fit the data.

(33) $$R-square=1-\frac{\sum_{i=1}^{n}w_{i}{\left({\hat{y}}_{i}-\bar{y_{i}}\right)}^{2}}{\sum_{i=1}^{n}w_{i}{\left(y_{i}-\bar{y_{i}}\right)}^{2}}$$

The statistical parameter of RMSE is also called the fitting standard deviation of the regression system. A good choice and fitting of the model contributes to successful data prediction.

(34) $$RMSE=\sqrt{\frac{1}{n}\sum_{i=1}^{n}w_{i}(y_{i}-{\hat{y}}_{i}{)}^{2}}$$

It can be seen that the prediction results in Figure 11a,c,d are very good, as their R^2^ values are all close to 1. Gumbel-copula produces the best prediction result and the smallest fitting standard deviation. However, for Clayton-copula in Figure 11b, the error is larger, especially for the low points in the curve. Generally speaking, the copula prediction model is more accurate. Although it does not achieve overwhelming superiority in accuracy over other methods (e.g., the neural network approach), its major advantage lies in its ability to make predictions much earlier. 

**Figure 12:**
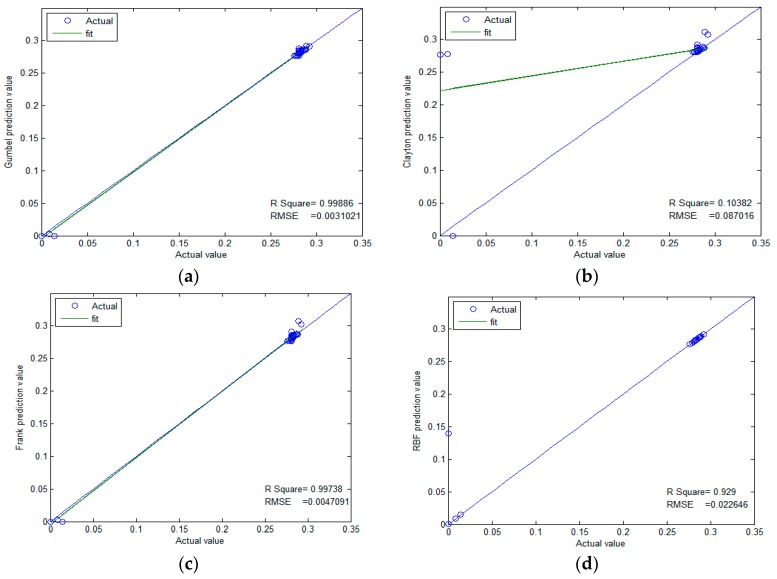
Comparison of correlation coefficients through four methods. (**a**) Gumbel-copula; (**b**) Clayton-copula; (**c**) Frank-copula; (**d**) RBF-ANN.

The results of the comparison are shown in Figure 12. Gumbel-copula is among the three models that has the highest correlation coefficient; Clayton-copula has some large error.

**Figure 13:**
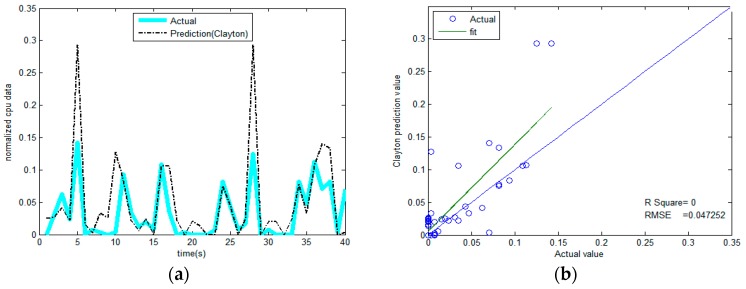
Prediction of CPU through flow. (**a**) Clayton-copula for CPU series; (**b**) Comparison correlation coefficient of CPU.

When the correlation of the two variables is not strong, the prediction accuracy will be significantly decreased. Figure 13a shows the prediction results of the CPU by flow data; we can see that the fit parameter (R^2^) tends to 0 from Figure 13b, indicating the presence of large errors, but it is accurate on the prediction of the peak, and the peak is our main focus. The result also shows that the current network behavior is more harmful to memory and a memory-consuming behavior.

#### Risk Assessment

In the experiment, we continued to employ the above data sets for the risk assessment. The time series included flow, CPU, and memory data. We used the GARCH-t (1,1) model to obtain the marginal distribution of each time series. The parameters obtained by the MATLAB toolbox are shown in Table 2, and the interpretations of the parameters are as described in Section 3.1.1.

**Figure 14:**
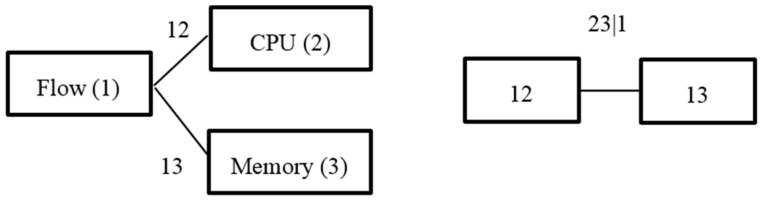
C-Vine decomposition of three variables.

Substituting parameters for the table into Equation (21), we obtain the condition of marginal distribution of each index. Since the flow has a certain impact on the CPU and memory, it can be set as a guide variable. Figure 13 describes the logical structure of several variables by C-Vine; each edge represents a pair copula connection functions, assuming that the code of the traffic, CPU, and memory are 1, 2, 3, respectively. C-Vine’s decomposition diagram is shown in Figure 14.

**Figure 15:**
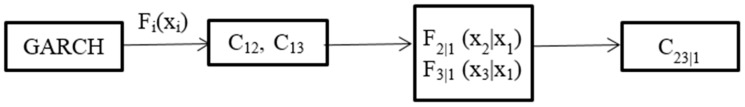
Estimation processes of parameters initial values.

Before the log maximum likelihood estimation of the copula pair density function, the initial values of the parameters in each tree are firstly processed, as shown in Figure 15. The results of estimation are presented in Table 3.

**Figure 16:**
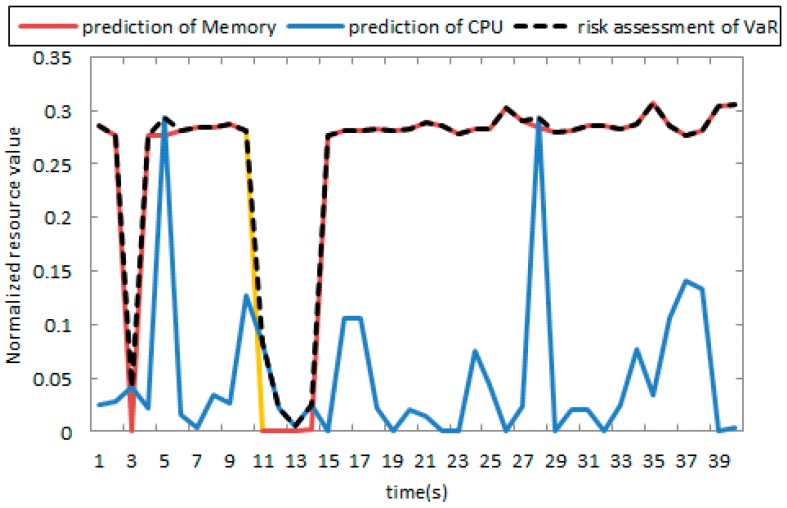
Prediction curve of the VaR.

We assumed that the shortages of each resource had the equal probability to appear. After all, the model parameters are obtained, and we combine them with Equation (16) and obtained the joint density function of the three resources. Then we calculated the risk based on Equation (23). At a given confidence level of 95%, the value of VaR was then calculated. When there is abnormal network behavior, the value of the VaR is far greater than that of the normal. Figure 16 shows the prediction of the VaR curve of the last 40 data points, and it can be seen that the curve covered the high point of the two resource sequence curves; when there is a peak, the curve can give an alarm.

### 4.2. Simulation of Incomplete Strategy of the Minority Game

The mainstream network simulation software OPNET (Version 14.5, Riverbed Technology Pte. Ltd., San Francisco, CA, USA)—with its three-layer modeling features, outstanding performance in a complicated environment, and realistic simulations of complex flow—provides great help in network design, construction, analysis, and management. To verify the validity of the model, we use OPNET simulation software to simulate the TCP flood attack and embed the defense model in the switch. During the first quarter of 2014, over 87% of DDoS attacks were focused on the network infrastructure, with TCP floods being one of the most popular attacks. Although there are many types of traffic attacks, the common feature of these attacks is repetitious transmission of certain types of packets. Due to the similarity among the transmission behaviors, we choose the most typical attack (TCP FLOOD) to simulate the model.

**Figure 17:**
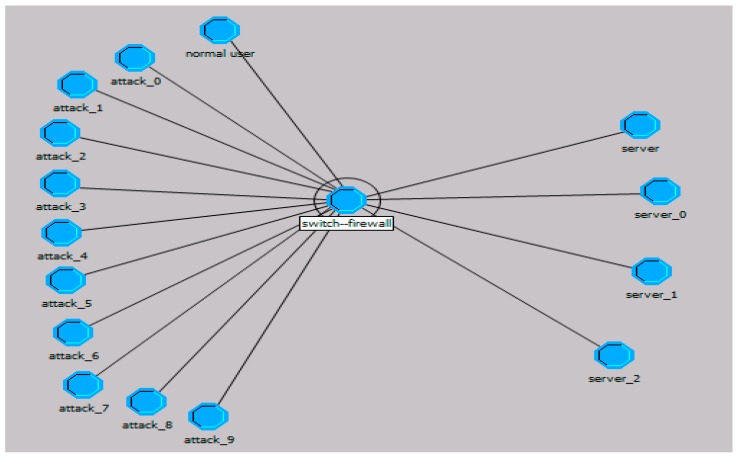
Incomplete minority game (IMG) model of the network topology map.

The model first built a link and packet formats, then created the nodes of the client, attack, server, and switch, constructing a network as shown in Figure 17. In the simulation, the network consists of 10 attacking hosts, one normal user, and three servers. Out of these servers, one server is a victim server. The proposed methodology is scalable, because DDoS attacks include much higher numbers of attackers. We can set the attack nodes to generate packets at the higher frequency. The network traffic that is received by a server is not constant, and has some peaks and some tails. The simulation scenario also includes legitimate network traffic from a simulated legitimate user.

All of the nodes start at 0 s to generate attack traffic and normal flow. Port values of each attack packet vary from 0 to 8, source addresses are randomly generated (greater than zero) and vary from 1 to 256, and both port number and source address is 0 for normal flows. The destination address of all the nodes is 7, which represents the server address under attack. The codes of the switch node forwarding packets and server node receiving an interrupt, especially game rules code are the keys of the model. Statistics are collected on a millisecond time interval.

The duration of simulation is 30 s, and defense measures are not running in the first 10 s. Our IMG model is started at the tenth second. Codes of the model focus on the preparation of the switch node, server node module, and the relevant programs of the game rules. Then, collection of the throughput and packet information of switch and other nodes is conducted.

It was already mentioned that the peak of a resource can be predicted through traffic resources. For simplicity, let us assume that the peak is proportional to a resource, and set the attractor value to 70% of the total resource capacity. The influence of the value of the attractor on the filtering of normal packets will be discussed below. If the flow rate exceeds this value, the server will crash. In OPNET, the corresponding value of the server’s maximum throughput is 1600 packets/s. Our aim is to protect the victim server from a DDoS attack, monitor the permeability of the normal flow through a firewall, and finally, provide an analysis of the data.

We performed simulation experiments for two different scenarios. We monitored the throughput from the remote network to the local network through a firewall (switch), and compared the results of two scenarios.

Scenario 1: In this set of experiments, we conducted simulations to compare IMG with the existing technique. All of the traffic packets are forwarded without any change and without putting the active defense on. At ten seconds, CAR (committed access rate) is employed. To keep the server working normally, flows in excess of the server’s maximum throughput processing are dropped directly. Changes of the throughput are recorded, and the permeability of the normal flow is tested.

Scenario 2: In this set of experiments, the active defense starts after ten seconds of the beginning of the attacks. The changes of the throughput are recorded. It is observed whether the flows are reduced to a reasonable range, and the permeability of the normal flows is tested. The results are compared with that of Scenario 1.

#### 4.2.1. Simulation Process for Scenario 1

In this section, active defense in the firewall (switch) does not start, and it just starts throttling once the threshold has been reached. CAR technology is used in the switch port. The technology specifies the traffic sent to the target through a random early dropping policy on upstream queues. Once the flows exceed a threshold, the packets beyond the threshold are discarded or delayed forward, irrespective of being legal or illegal. In this way, it makes the throughput to the server drop below the threshold.

**Figure 18:**
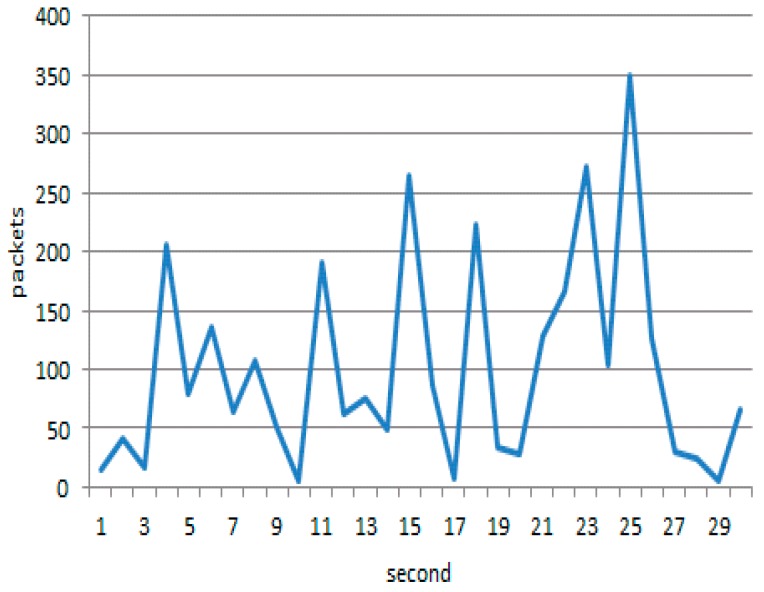
Background flows from normal user.

**Figure 19:**
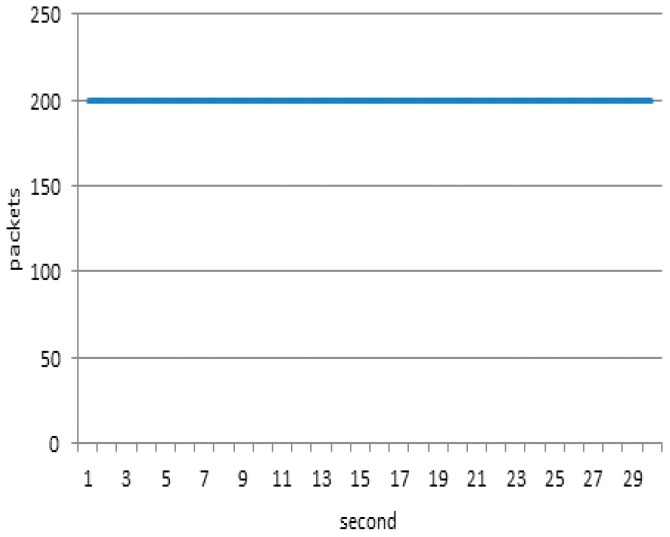
Flows of attack from Attacker 1.

Firstly, network traffic was monitored under heavy load. The attack nodes generate packets at a certain time interval, which follows the constant statistical distribution of 0.005. The rate of normal node-generating packets is subjected to a Poisson distribution. Obviously, the attack nodes generate a higher volume of traffic than normal nodes, as depicted in Figure 18 and Figure 19.

**Figure 20:**
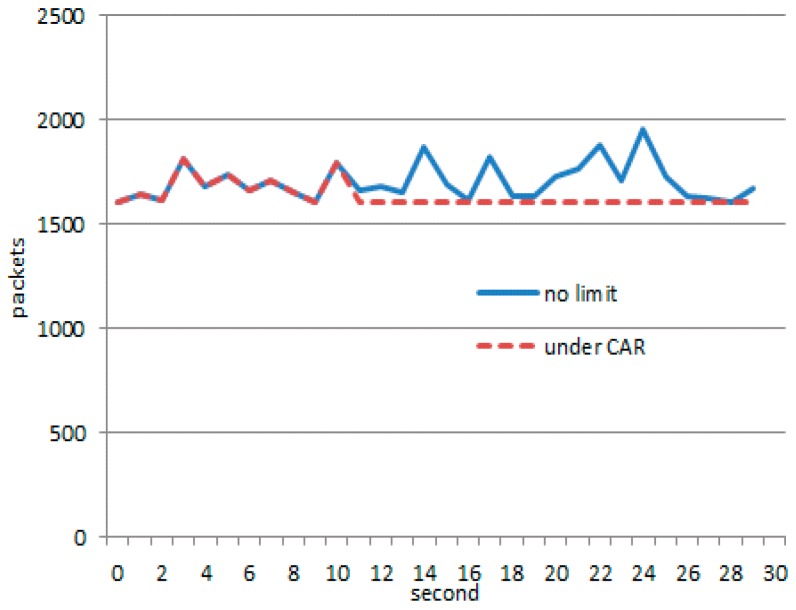
Throughput to the server under committed access rate (CAR) (30 s).

When defense system (CAR) starts, network bandwidth rapidly drops to 1600 packets/second, and the link between the gateway and the server maintains a high level of throughput, as depicted in Figure 20. If there is no defense, the throughput will quickly exceed the threshold (1600 packets/second). This can lead to a server crash. Although the CAR method makes bandwidth fall to the threshold, the number of normal packets reaching the server are very small. Only 271 packets reached the server, whereas, the normal node sent 3012 packets.

#### 4.2.2. Simulation Process for Scenario 2

**Figure 21:**
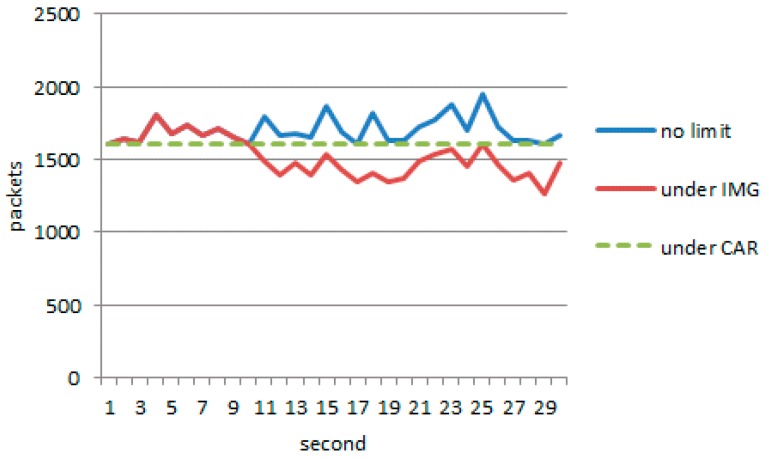
The throughput to the server under IMG (30 s).

When using an IMG model, attack nodes and normal nodes remain working as before. However, active defense starts at the tenth second. Obvious deviation is shown in Figure 21. It can be observed that the throughput falls significantly, and has been hovering in a narrow range of 1600–1200. Especially, the number of normal packets reaching the server amounts to 2054.

However, it cannot be denied that there is some noise from similar packets. These packets can be randomly generated by attack nodes. Similar packets have characteristics similar to normal packets. For example, packets with the same ports or with the same source address have similar characteristics. Thus, the active defense treats them as the minority.

**Figure 22:**
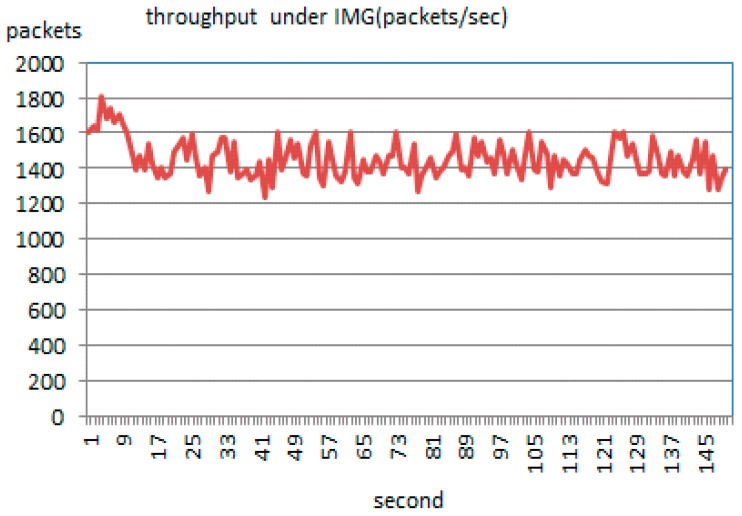
Throughput to the server under IMG (150 s).

Figure 22 represents the result of the simulation running continuously for 150 s. It accomplishes the same goals of depressing the flows and permeability.

**Figure 23:**
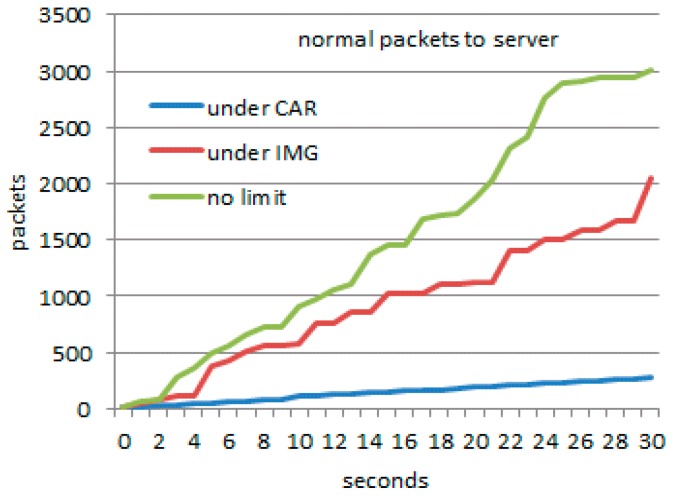
Comparison of the legit packets transmitted under CAR /IMG/No limit.

As can be seen from Figure 23, with the continuous game, the proportion of legal packets through the IMG method is much higher than the proportion of CAR, which achieves the goal of trying to protect the legitimate link. When the system is going to collapse, even the normal traffic should be suppressed. Source IP addresses as the destination IP address, port numbers, and protocols are the only parameters needed for the suppression algorithm. Our goal is to give priority to ensuring that the server does not crash.

It is inevitable that IMG may destroy some legitimate packets. This method exerts more influence on the majority links than the minority ones, but it achieved quick protection of the server (to avoid collapse) and—As much as possible—Improved the legal package pass rate, so it is worthwhile.

**Figure 24:**
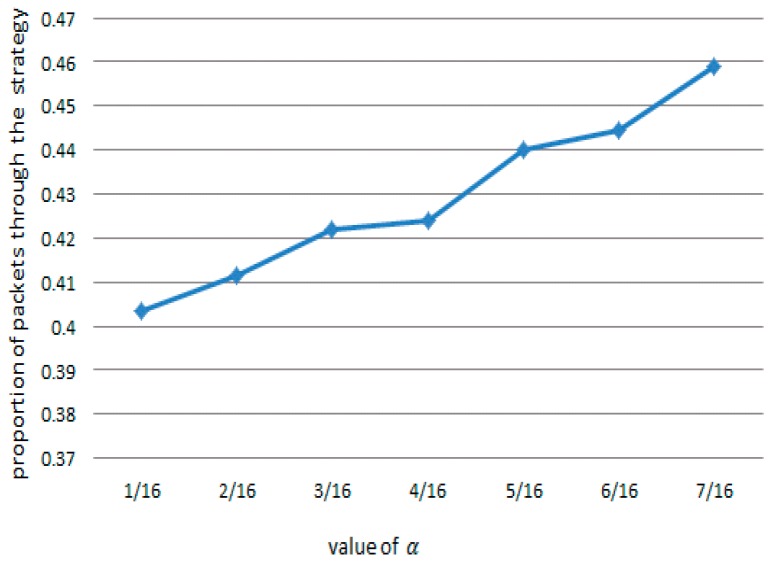
The relation between α and proportion of selected packets.

In what follows, we will discuss the influence of the attractor on the filtering results. Firstly, the relationship between the value of α and the proportion of the selected packets is illustrated. In fact, both the normal and attacking packets are likely to be chosen during each round of selection, and a higher value of α means that more packets meet the condition and more time is needed to decide on, and forward, the packets. In Scenario 2, we set the value of α from 1/16 to 7/16, and then compute the ratio of the number of packets chosen to be transmitted in 30 s to the total number of transmitted packets. It can be seen from Table 4 that when the value of α increases, the proportion of normal packets that can penetrate the server also increases, and the simulation takes more time. Figure 24 shows the proportion of the normal packets that penetrate the server as a function of α.

**Figure 25:**
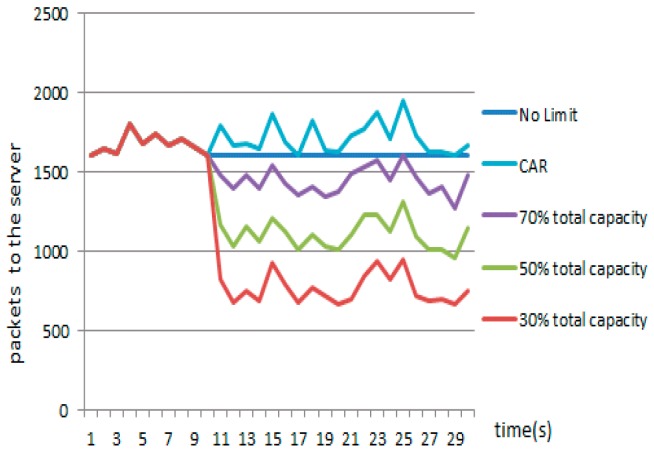
Comparison of the throughput under CAR/IMG.

**Figure 26:**
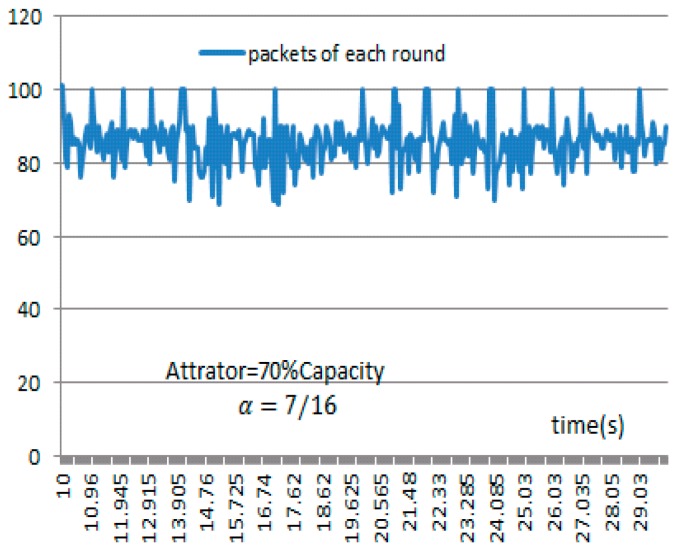
IMG packets of each round.

The value of the attractor determines the upper bound of the balance point in the IMG method. Figure 25 shows the results achieved in Scenario 2 when we set the value of α to 7/16 and the value of attractor to 70%, 50%, and 30% of the network throughput, respectively. We also compare the curve obtained under the CAR constraint with the curve obtained without the CAR constraint. Figure 26 shows that in IMG, the flow quickly decreases to the attractor and then hovers around that level. Table 5 compares the number of normal packets that penetrate the server with varying attractors. The higher the attractor, the higher the bandwidth capacity, and more normal packets pass. Figure 25 shows the results achieved after each round of IMG gaming when the attractor is set to 70% of the network throughput and α is set to 7/16, where the horizontal coordinate denotes the time stamp of each round of gaming. It can be seen that the number of packets chosen during each round ranges from 69 to 101. Analysis of the tables and figure above indicates that although both IMG and CAR can control flows stably, the number of effective packets that pass differs greatly. In addition to controlling flows, IMG offers better support to penetration of normal packets and gives expected protection to important node resources.

Undeniably, normal packets are likely to be abandoned. The following three considerations are taken into account: (1) normal packets may be retransmitted in the case of timeout. An example is the 200 ms retransmission scheme in TCP. (2) From the experimental results, it can be seen that the normal packets pass the defense module at a probability of 68%; that is, most of the normal packets can pass, while most of the attacking packets are intercepted. (3) In the case of an attack, the task of preventing the entire system from collapse takes priority over the task of maximizing the probability that the normal packets pass.

In the experiment, we emphasize the ratio between attack traffic and normal traffic. The model performance in a real application should be considered. To go along with this, we modify the program and reset parameters in the OPNET simulation platform. The send rate of normal packets follows the Poisson distribution (mean value is set to 0.005), and that of attack packets follows the constant statistical distribution of 0.00006. The average traffic is 133,400 packets per second. We obtain similar results, which show that the proportion of legal packets through the IMG method is still higher than the proportion of CAR.

## 5. Conclusions

In this paper, we proposed an active defense model focused on protecting the resources under the tolerant invasion. In the proposed model, we firstly predict the peak of related resources through the traffic information at the firewall. Whenever there is an alert, the firewall starts the active defense of an incomplete strategy of the minority game. To prove the validity and applicability of the proposed model, results are simulated using OPNET. The simulation results show that this method implements the suppression of attack flows, improves the permeability of the normal flows, and protects the safety of the target node. In particular, it is a universal defense method that does not require distinguishing of the DDoS attack flows and normal peak flows.

The module of the proposed model can run separately, and can be used as an additional function added in firewalls, routers, and other network equipment. It can protect any IP node under the condition of low cost and has the advantage of convenient deployment. However, resource alerts and active defense is embedded in the firewall, and their calculation process leads to a time delay. The study of time delay is not properly addressed in this model, but the game time delay does not obviously affect the propagation velocity of packets in the viewpoint of the OPNET simulation results. In the future work, we expect to undertake quantified analysis to the game time delay and raise the permeability of the normal flows.

## Figures and Tables

**Table 1:** Incomplete strategies and history information.

Strategy_n−2_	Strategy_n−1_	State_n−2_	State_n−1_	Current Scenarios Estimate	Choice
0	0	0	0	Never send or choose not to send in the first couple of steps, did not affect the resources, could be estimated as the normal flows.	1
0	0	0	1	Never send or choose not to send in the first couple of steps, affected the resources, could be estimated as the attack flows.	0
0	0	1	0	Never send or choose not to send in the first couple of steps, affected the resources, could be estimated as the attack flows.	0
0	0	1	1	Never send or choose not to send in the first couple of steps, affected the resources, could be estimated as the attack flows.	0
0	1	0	0	Choose to send in the previous step, did not affect the resources, could be estimated as the normal flows.	1
0	1	0	1	Choose to send in the previous step, affect the resources, could be estimated as the attack flows.	0
0	1	1	0	Choose not to send in the first couple of steps, affect the resources, choose to send in the previous step, did not affect the resources, could be estimated as the normal flows.	1
0	1	1	1	Choose not to send in the first couple of steps, affect the resources, choose to send in the previous step, affect the resources, could be estimated as the attack flows.	0
1	0	0	0	Choose to send in the first couple of steps, did not affect the resources, choose not to send in the previous step, did not affect the resources, could be estimated as the normal flows.	1
1	0	0	1	Choose to send in the first couple of steps, did not affect the resources, choose not to send in the previous step, affect the resources, could be estimated as the normal flows.	1
1	0	1	0	Choose to send in the first couple of steps, affect the resources, choose not to send in the previous step, did not affect the resources, could be estimated as the attack flows.	0
1	0	1	1	Choose to send in the first couple of steps, affect the resources, choose not to send in the previous step, affect the resources, could be estimated as the attack flows.	0
1	1	0	0	Choose to send in the first couple of steps, did not affect the resources, could be estimated as the normal flows.	1
1	1	0	1	Choose to send in the first couple of steps, did not affect the resources, could be estimated as the attack flows.	0
1	1	1	0	Choose to send in the first couple of steps, affect the resources, but did not affect in the first step, could be estimated as the normal flows.	1
1	1	1	1	Choose to send in the first couple of steps, all affected resources, but did not affect in the first step, could be estimated as the attack flows.	0

**Table 2:** The parameters’ values of estimation.

Parameters	Flow	CPU	Memory
$c_{0}$	–0.0821	0.0055	–0.0570
$c_{1}$	–0.3954	–0.3412	–0.4276
ω	–0.3523	–0.2012	–0.4752
α	0.3720	0.1495	0.1785
β	0.4278	0.5000	0.3352
γ	0.5722	0.5000	0.5973
v	3.0958	3.0703	6.0349
λ	–0.2036	0.0209	–0.2782

**Table 3:** The initial values of estimation.

Parameters	$c_{12}$	$c_{13}$	$c_{23|1}$
The initial parameter values	0.0793	0.2351	0.1323

**Table 4:** Packets and time statistics for the experiment in different α.

α Value	Simulation Time (Seconds)	IMG (Packets) (Normal Packets Arriving to Server)
7/16	45 s	2054
6/16	43 s	1990
5/16	40 s	1970
4/16	38 s	1898
3/16	35 s	1842
2/16	35 s	1842
1/16	34 s	1806

**Table 5:** Packets arriving to server statistics for the experiment in different Attractor.

Attractor Value	IMG (Packets) (Normal Packets Arriving to Server)
70% capacity	2054
50% capacity	1559
30% capacity	1119
CAR	271
No limit	3012
